# Factor XIII and Fibrin Clot Properties in Acute Venous Thromboembolism

**DOI:** 10.3390/ijms22041607

**Published:** 2021-02-05

**Authors:** Michał Ząbczyk, Joanna Natorska, Anetta Undas

**Affiliations:** 1 John Paul II Hospital, 31-202 Kraków, Poland; michalzabczyk@op.pl (M.Z.); j.natorska@szpitaljp2.krakow.pl (J.N.); 2 Institute of Cardiology, Jagiellonian University Medical College, 31-202 Kraków, Poland

**Keywords:** acute thrombosis, coagulation, factor XIII, fibrin clot, venous thromboembolism

## Abstract

Coagulation factor XIII (FXIII) is converted by thrombin into its active form, FXIIIa, which crosslinks fibrin fibers, rendering clots more stable and resistant to degradation. FXIII affects fibrin clot structure and function leading to a more prothrombotic phenotype with denser networks, characterizing patients at risk of venous thromboembolism (VTE). Mechanisms regulating FXIII activation and its impact on fibrin structure in patients with acute VTE encompassing pulmonary embolism (PE) or deep vein thrombosis (DVT) are poorly elucidated. Reduced circulating FXIII levels in acute PE were reported over 20 years ago. Similar observations indicating decreased FXIII plasma activity and antigen levels have been made in acute PE and DVT with their subsequent increase after several weeks since the index event. Plasma fibrin clot proteome analysis confirms that clot-bound FXIII amounts associated with plasma FXIII activity are decreased in acute VTE. Reduced FXIII activity has been associated with impaired clot permeability and hypofibrinolysis in acute PE. The current review presents available studies on the role of FXIII in the modulation of fibrin clot properties during acute PE or DVT and following these events. Better understanding of FXIII’s involvement in the pathophysiology of acute VTE might help to improve current therapeutic strategies in patients with acute VTE.

## 1. Introduction

Factor (F) XIII, a fibrin stabilizing factor, is a 325 kDa protransglutaminase representing the transglutaminase-like superfamily, which involves calcium-dependent enzymes leading to post-translational modifications of proteins and generation of isopeptide bonds resistant to proteolytic degradation [1]. FXIII is present in cells, including monocytes, osteoblasts, and megakaryocytes, and in plasma [2]. Both forms of FXIII are involved in blood coagulation [2]. FXIII contains two catalytic A subunits (about 83 kDa) and two noncatalytic (inhibitory) B subunits (about 80 kDa) that form heterotetramers (FXIII-A_2_B_2_) [3]. FXIII-A_2_B_2_ binds to fibrinogen residues γ390-396 via the B subunits with a high affinity (a dissociation constant up to 10 nM) [4,5]. As shown in mice homozygous for the fibrinogen γ-chain mutation (Fibγ390-396A), this change is associated with reduced binding of FXIII-A_2_B_2_ to fibrin(ogen), while delayed FXIII activation and slower formation of fibrin γ- and α-chain crosslinks were observed in plasma [6]. Platelet FXIII-A_2_ is derived mainly from megakaryocytes or synthesized de novo and its concentrations are relatively high, in the range from 46 to 82 femtograms per platelet [7,8]. After platelet activation, FXIII-A_2_ is exposed on the platelet surface [9]. Physiologically, FXIII-B is synthesized in the liver in excess, and about 50% of this subunit circulates in plasma and can bind fibrinogen in the absence of FXIII-A_2_ [4]. Only 1% of FXIII-A_2_ is estimated to circulate as a free form [5]. The B subunit protects the A subunit from spontaneous proteolysis and therefore prolongs its circulating half-life [10]. FXIII circulates in blood at concentrations between 14 and 28 (average 22) mg/L and has a half-life of 9–14 days [11].

In the presence of fibrin, thrombin converts FXIII to its activated form (FXIIIa) by cleavage of FXIII-A at Arg37-Gly38 and release of an activation peptide, followed by Ca^2+^-driven dissociation of FXIII-A and B subunits [3]. Activation of platelet FXIII-A_2_ occurs after thrombin-mediated cleavage of the activation peptides [12] or at high calcium concentrations [13]. FXIIIa catalyzes the formation of intermolecular bonds not only between fibrin monomers but also between α2-antiplasmin, fibronectin, vitronectin, thrombospondin, and collagen [14]. FXIII is essential for maintaining hemostasis, including the mechanical stabilization of a fibrin clot and the protection of newly formed fibrin clots from fibrinolysis at the site of vascular injury.

Hemostatic relevance of a normal activity of FXIII is substantiated by the severe bleeding diathesis resulting from FXIII deficiency with the prevalence of 1 case per 2 million [11,15]. Mild acquired FXIII deficiencies characterized by FXIII levels above 30% of normal plasma concentrations can occur due to consumption or decreased synthesis of FXIII observed in patients with autoimmune conditions, due to excessive consumption in thrombotic states or impaired synthesis in liver diseases or leukemia [16,17].

It has been suggested that FXIII may exert antithrombotic effects at least in part by lowering platelet adhesion to fibrin [18]. Furthermore, FXIII is involved in wound healing by crosslinking extracellular matrix proteins and fibrin [18,19], has a proangiogenic effect [20], and modulates inflammation/infection due to promoting cellular signaling between leukocytes and endothelial cells [21].

FXIII plays a critical role in crosslinking of extracellular matrix proteins, such as fibronectin, collagen or von Willebrand factor [18], which leads to FXIII-A deposition, influences cell-matrix interactions, and alters the properties of fibrin clots [22]. Increased crosslinking by FXIII is associated with enhanced stiffness of fibrin network, which can impact the ability of cells, including endothelial cells, to thrombus remodeling [22]. Moreover, kinetics of fibrin fiber crosslinking may impact the structure and properties of extracellular matrix, which have been suggested to regulate cell behavior and tissue-clot interactions [23]. FXIII as the key determinant of thrombus stiffness and stability can influence the response of endothelial and blood cells to mechanical stimuli [24,25]. However, the impact of functional and mechanical properties of crosslinked fibrin on thrombus remodeling and its interaction with cells in vivo require further studies including use of three-dimensional in vitro models of fibrin clots.

Fibrin following the action of FXIIIa ensures not only clot stability but also its resistance to enzymatic lysis. The catalytic half-life of FXIIIa was established in an animal model of pulmonary embolism (PE). It has been found that after about 20 min FXIIIa activity within thrombi decreased to 50%, suggesting the presence of mechanisms leading to local FXIIIa inactivation [26]. Since in vivo specific FXIIIa inhibitors are unknown, mechanisms such as proteolytic cleavage by thrombin [27] or by proteolytic enzymes of polymorphonuclear cells have been proposed to inactivate FXIIIa [28].

## 2. Genetic Variants of FXIII

The FXIII-A subunit is encoded by a gene composed of 15 exons and 14 introns located on chromosome 6p24–25, while the FXIII-B subunit gene on chromosome 1q31–32.1 contains 12 exons and 11 introns [29]. Several polymorphisms, mostly in non-coding regions, have been described in the FXIII-A subunit gene. Among common FXIII-A subunit gene polymorphisms, much attention has been paid to the p.Val34Leu variant, which occurs in about 25% of Europeans [30]. The FXIII 34Leu compared with FXIII 34Val is associated with about 2.5-fold faster FXIII activation and fibrin crosslinking [31]. Faster activation of FXIII in general results in the formation of clots with smaller pores and thinner fibers. However, this effect depends on fibrinogen concentrations. Fibrin clots prepared from plasma samples of subjects homozygous for FXIII 34Leu compared to FXIII 34Val were characterized by thinner fibrin fibers and reduced clot permeability at normal fibrinogen levels. At high fibrinogen levels thicker fibrin fibers were formed, resulting in increased clot permeability and susceptibility to lysis in subjects homozygous for FXIII 34Leu compared to FXIII 34Val [3,31,32,33].

Regarding the FXIII-B gene polymorphisms, p.His95Arg polymorphism increases the risk of stroke and reduces the risk of myocardial infarction [34], while VS11, c.1952 + 144 C>G (Intron K), polymorphism lowers the risk of coronary atherosclerosis and myocardial infarction [32,35].

## 3. FXIII as a Modulator of Fibrin Clot Properties

FXIII is the key determinant of thrombus mechanical and biochemical stability [36]. FXIIIa crosslinks glutamine and lysine residues in the α- and γ-chains of fibrin monomers by forming covalent isopeptide bonds [36]. This results in formation of γ-γ dimers as well as α-α and γ-α polymers leading to increased clot stiffness and its stabilization, as evidenced using recombinant fibrinogens in vitro [37]. It has been shown that crosslinking by FXIII decreases the elasticity of fibrin fibers and increases fibrin elastic modulus (stiffness), and that crosslinking by FXIIIa of other plasma proteins to fibrin modulates fibrin clot properties [38]. FXIIIa has also an ability to bind α2-antiplasmin to fibrin, which strongly inhibits plasmin generation assessed by measuring a tissue plasminogen activator concentration required for 50% lysis of clots prepared from normal or α2-antiplasmin-deficient plasma in the presence or absence of FXIIIa inhibitor [39].

Hethershaw et al. [40] showed, for the first time, in a purified fibrinogen model that FXIII exerts a direct effect on the fibrin network structure. Clot structure results not only from a fibrinogen concentration, which is the most abundant protein within the plasma fibrin clot (about 70% of the clot mass), or fibrinogen function [41], but also from the amount and activity of other proteins bound to fibrin, including fibronectin (13% of the clot mass), α2-antiplasmin (2.3% of the clot mass), complement component C3 (1.2% of the clot mass), FXIII (1.2% of the clot mass), and prothrombin or antithrombin (both below 0.5% of the clot mass) [42]. Clots formed in vitro from human fibrinogen in the presence of FXIII had 2.1-fold reduced fibrin clot permeability (K_s_) compared to fibrin clots formed in the absence of FXIII, along with 12.2% increased fibrin fiber density assessed by scanning electron microscopy (SEM) [40]. Moreover, SEM revealed that fibers in clots formed in the presence of FXIII were 15% thinner compared to clots prepared without FXIII [40]. In vitro addition of purified human FXIII to human fibrinogen was also associated with increased resistance to fibrinolysis, reflected by about 16% prolonged lysis time of clots formed in the presence of FXIII [40]. Therefore, FXIIIa could be a potential target in therapy of thromboembolic diseases [6]. Rijken et al. [39] have also shown that about 31% of α2-antiplasmin remained within the clot stabilized by FXIIIa after its in vitro compaction by centrifugation, while only 4% remained without the FXIIIa due to non-covalent interaction of α2-antiplasmin with fibrin. Moreover, FXIIIa crosslinks to fibrin thrombin activatable fibrinolysis inhibitor (TAFI), a fibrinolysis inhibitor, that contains specific acyl acceptor and acyl donor residues, and glycine residues at positions 2, 5, and 294 are preferred acyl donor sites for FXIIIa [43]. An influence of histones, released during neutrophil extracellular trap (NET) formation, on fibrinolysis and its association with FXIII has been recently studied by Locke et al. [44]. They have shown that histones, which are rich in lysine residues, competitively inhibit plasmin to delay fibrinolysis in the in vitro model [44]. This effect was enhanced by covalent crosslinking of histones to fibrin, in a FXIIIa-dependent manner. The FXIIIa inhibitor (T101) is able to block such interaction and was suggested as a potential antithrombotic treatment in acute thrombosis [44]. A similar effect was achieved using low-molecular-weight heparin (LMWH) to inhibit the histone-fibrin crosslinking and improve fibrinolysis [44]. Recently, a novel specific inhibitor, ZED3197, has been described as a potential drug candidate in anticoagulation targeting FXIIIa for at least short-term therapy to modulate clot structure and enhance fibrinolysis [45]. ZED3197 is a potent and selective peptidomimetic inhibitor of FXIIIa, covalently and irreversibly binding FXIIIa [45]. However, clinical studies are needed to corroborate the therapeutic strategy based on modulation of FXIIIa.

## 4. Role of FXIII in Venous Thromboembolism (VTE)

It has been hypothesized that FXIII levels and/or activity are associated with the manifestation and severity of acute VTE, mainly due to the modulating effect of FXIII on fibrin meshwork [3]. In a nested case-control study involving 21,860 participants, including 462 patients who developed VTE, the overall risk of first VTE event was not associated with the FXIII-A subunit antigenic levels (odds ratio (OR) = 1.1, 95% confidence interval (CI) 0.8–1.6 for 5th vs. 1st quintile of plasma FXIII level) [46]. Mezei et al. [47] have shown in a cohort of 218 VTE patients (women, 52%) compared to age- and sex-matched controls that three months after the acute event, FXIII antigen levels and activity were higher in female patients. The authors reported that FXIII antigen levels in the upper tertile were associated with 2.5-fold higher risk (95% CI 1.18–5.38) of VTE, while elevated FXIII-B antigen levels reduced the VTE risk solely in men (OR = 0.19, 95% CI 0.08–0.46) [47].

Available data indicate that FXIII levels/activity are not associated with the risk of recurrent VTE. No differences in plasma FXIII levels were observed among 11 patients with recurrent VTE and 33 non-recurrent VTE subjects in the study by Baker et al. [48], which was associated with no differences found in fibrin clot structure or fibrinolysis rates.

There is robust evidence that the p.Val34Leu allele protects against VTE as well as against myocardial infarction [33]. Wells et al. [33] showed in a meta-analysis of 12 studies with genotyping for FXIII p.Val34Leu allele (3165 patients diagnosed with VTE and 4909 controls) a small but significant protective effect of this polymorphism against VTE (OR = 0.63, 95% CI 0.46–0.86 for the Leu/Leu homozygotes; OR = 0.89, 95% CI 0.80–0.99 for the Leu/Val heterozygotes; and OR = 0.85, 95% CI 0.77–0.95 for the homozygotes and heterozygotes combined). The protective effect of FXIII p.Val34Leu allele against VTE (OR = 0.80, 95% CI 0.68–0.94, *p* = 0.007) was confirmed by Gohil et al. [49], who compared carriers of the Leu allele (Leu/Leu + Leu/Val) against wild-type (Val/Val) in a meta-analysis involving 173 case-control analyses of about 120,000 cases and 180,000 controls. Mechanisms between this protection are complex and unclear. It has been shown that increased FXIII activation in 34Leu carriers may result in ineffective crosslinking and facilitated fibrin degradation [32]. Moreover, it has been observed that FXIII 34Leu allele accelerates not only thrombin-mediated FXIII-A cleavage, but also increases by about 40% γ-γ-dimer formation at the site of microvascular injury in healthy individuals heterozygous for the 34Leu allele compared to those homozygous for the 34Val allele [50]. This effect was abolished by oral anticoagulation with vitamin K antagonists [50]. In contrast, the FXIII p.Val34Leu polymorphism (both for Val34Leu or Leu34Leu vs. Val34Val) has failed to be associated with cancer-related VTE in the prospective Vienna Cancer and Thrombosis Study [51]. Moreover, several mutations have been shown to accelerate (e.g., p.Val34Leu, p.Val34Met) or reduce (e.g., p.Gly33Ala, p.Val34Ala, p.Val29Ala) FXIII activation rates in a murine model of thrombosis [52]. The FXIII variants associated with increased activation rates of FXIII led to enhanced fibrin crosslinking, which, however, had no impact on thrombus size [52]. In conclusion, other FXIII-A polymorphisms have not been shown to be linked with VTE risk. Regarding the FXIII-B gene polymorphisms, p.His95Arg and VS11, c.1952 + 144 C>G (Intron K), have not been associated with VTE [34,47].

### 4.1. FXIII in Patients with Acute VTE

There is evidence that acute VTE events are associated with a transient decrease in FXIII levels in circulating blood. In 1986, Kłoczko et al. [53] showed in 19 acute deep vein thrombosis (DVT) patients that both FXIII activity and FXIII-A levels were reduced and concluded that FXIII levels returned to normal values within two weeks since the index event. Kool et al. [54] have reported that FXIII consumption in acute symptomatic DVT patients (*n* = 134) compared to age- and sex-matched controls in whom DVT was excluded (*n* = 171) was associated with about 20% lower FXIII-A subunit levels, but not with the levels of FXIII activation peptide. Increasing ORs for patients with FXIII-A subunit levels within the 4th (OR = 2.86, 95% CI 1.04–7.86) to 1st (OR = 7.74, 95% CI 3.04–19.74) quintiles suggested a dose-dependent association between FXIII-A subunit levels and the probability of having DVT [54].

**Figure 1:**
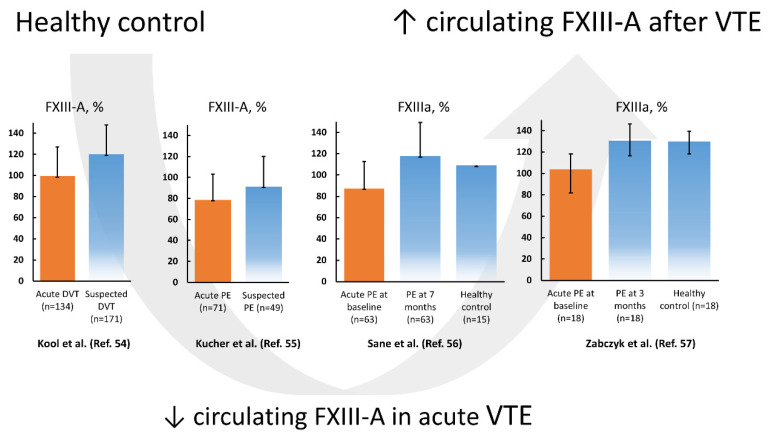
Factor XIII subunit A (FXIII-A) levels and FXIII activity (activated factor XIII (FXIIIa)) according to the literature data reported in venous thromboembolism (VTE), encompassing deep vein thrombosis (DVT) and pulmonary embolism (PE), on admission (orange bar) and after several weeks compared to controls (blue bars). Data regarding references [54,55,56] are presented as mean and standard deviation, while data from reference [57] is presented as median and interquartile range.

In 2003, Kucher et al. [55] showed in 71 acute PE patients that the circulating FXIII-A antigen level but not the subunit B level was decreased by 13.9% compared to 49 patients in whom PE was suspected but excluded. In that study the FXIII antigen level decreased with higher rates of pulmonary artery occlusion, along with reduced fibrinogen concentrations and elevated plasma D-dimer levels, suggesting coagulation activation and consumption of FXIII during massive thrombus burden [55]. The risk of PE increased several times (95% CI 1.4–35.3) in patients with FXIII-A subunit levels below 60% [55]. The authors concluded that reduced FXIII levels in acute PE can result from consumption of blood coagulation factors, including FXIII, within thrombi occluding the pulmonary arteries [55]. The concept of FXIII consumption was confirmed in non-high risk acute PE patients without any initial treatment (*n* = 35) and in those receiving LMWH (*n* = 28), in which FXIIIa level increased by 30% after a 7-month follow-up [56]. A drop in plasma FXIII activity from about 130 to 104% was also observed in 18 normotensive, non-cancer acute PE patients assessed on admission before initial treatment compared to age- and sex-matched controls [57]. After 3-month anticoagulant treatment with rivaroxaban, FXIII activity returned to levels observed in controls [57]. Based on available studies, lower FXIII activity and antigen levels are associated with the acute phase of VTE, followed by normalization during several weeks (Figure 1). The drop of FXIII during acute VTE suggests its consumption and accumulation within the thrombi, but the mechanism involved in such reduction and its potential role in prediction of clinical outcomes have not been established yet.

**Figure 2:**
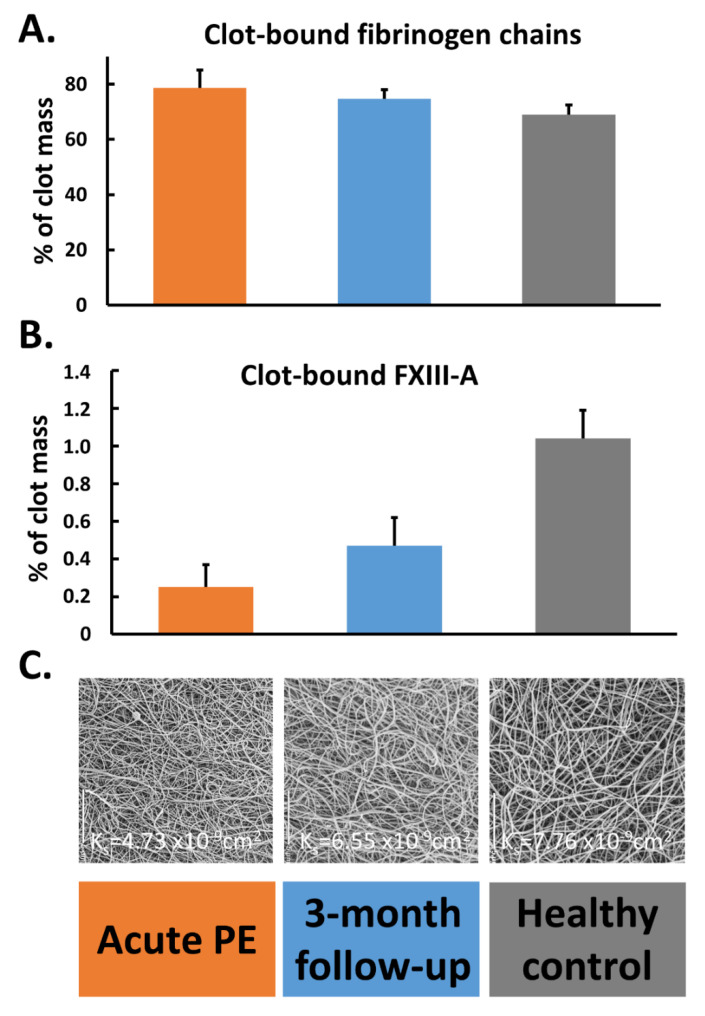
Percentage of clot-bound fibrinogen chains (panel (**A**)) and factor (F) XIII-A (panel (**B**)) along with scanning electron microscopy images (panel (**C**)) representing fibrin clot morphology reflected by clot permeability (K_s_) assessed during acute phase of pulmonary embolism (PE), after 3-month follow-up, and in healthy subjects. Data are presented as mean and standard deviation.

**Figure 3:**
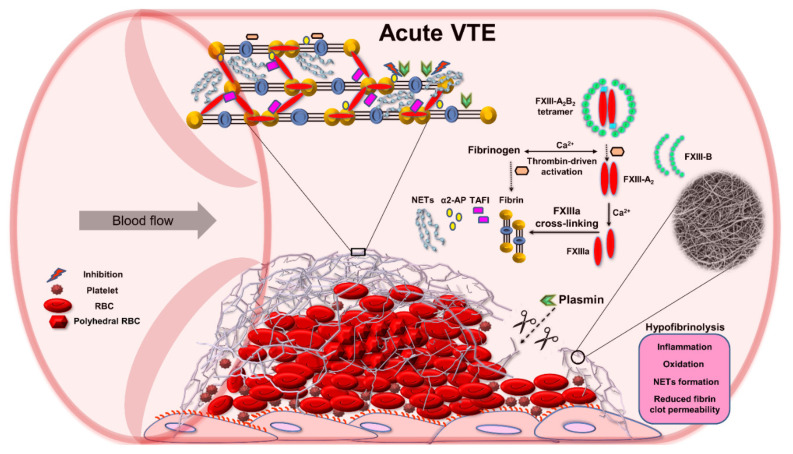
Role of factor (F) XIII in impaired fibrinolysis during acute venous thromboembolism (VTE). Abbreviations: α2-AP, alpha 2-antiplasmin; FXIIIa, activated factor XIII; NETs, neutrophil extracellular traps; RBC, red blood cell; TAFI, thrombin activatable fibrinolysis inhibitor.

A quantitative proteomics of fibrin clots prepared from citrated plasma subjected to endoproteinase LysC and trypsin-mediated protein digestion has shown that clots of acute PE patients differed from clots of control subjects in regard to about 200 proteins, including about 14% higher amount of clot-bound fibrinogen chains and 75% reduced amounts of clot-bound FXIII-A in PE patients (Figure 2A,B) [58]. It should be highlighted that a plasma clot model reflects a post-thrombotic state in patients in whom blood coagulation factors were already consumed in vivo [57]. Clots characterized by reduced FXIII amounts may differ with regard to other protein amounts, which can influence fibrin clot properties. Moreover, lower plasma activity of FXIII in PE patients assessed on admission compared to 3-month anticoagulant treatment with rivaroxaban was associated with 36% reduced clot-bound amounts of FXIII-A and prothrombotic fibrin clot properties, as evidenced by reduced K_s_ and prolonged clot lysis time (Figure 2B,C) [57]. No difference in amounts of clot-bound fibrinogen chains was found between fibrin clots of acute PE patients compared to those at the 3-month follow-up (Figure 2A). Of note, at 3 months, clot-bound amounts of FXIII-A were still about 50% lower than in age- and sex-matched healthy subjects [57]. Despite lower clot-bound amounts of crosslinking FXIII, the plasma fibrin clot phenotype of acute PE patients was more prothrombotic compared to that determined at the 3-month follow-up. Such impaired fibrin clot phenotype was associated with increased thrombin formation, enhanced inflammatory state, and potentially oxidative modifications of coagulation proteins. As was previously shown in acute PE patients, increased thrombin generation, inflammation, NET generation reflected by increased levels of citrullinated histones H3, and hypoxia are factors, which unfavorably modulate fibrin clot properties [59,60], regardless of FXIII consumption. On the other hand, it has recently been shown in vitro by Vasilyeva et al. [61] that purified human FXIII undergoes an extensive oxidation on methionine residues as a reactive oxygen species scavenger and protects other amino acids and/or proteins against oxidative modifications. However, the activity of oxidation-modified FXIIIa decreases significantly, which may influence fibrin clot properties (Figure 3). To our knowledge, this issue has not been investigated yet.

Growing evidence indicates that thrombus stability determines its susceptibility to embolization. Patients with a history of PE were characterized by less compact clot structure with higher susceptibility to lysis compared to patients following DVT episode [62], suggesting that a specific fibrin clot structure might contribute to clot fragmentation [36]. Moreover, less prothrombotic fibrin clot features may enhance breaking off large parts of venous thrombi, which predisposed mostly to central PE [63]. In line with this observation, it has been reported that mice with provoked DVT supplemented with FXIII formed more stable thrombi resistant to lysis, which reduced PE risk [64].

Taken together, FXIII appears to be of key importance in manifestations of acute VTE, affecting the likelihood of acute PE [65].

### 4.2. Drug Induced Effects on FXIII and Fibrin Properties

It has been demonstrated that non-vitamin K antagonist oral anticoagulants (NOACs) did not influence FXIII crosslinking. Varin et al. showed increased fibrin clot porosity and lysability using platelet-poor plasma spiked with rivaroxaban at peak concentrations encountered in vivo but this effect was not due to reduced FXIII activation [66]. Carter et al. showed that an addition of rivaroxaban or apixaban to normal plasma at maximum concentrations reported in plasma did not affect FXIIIa-mediated fibrin crosslinking or nanostructure of plasma clots formed in the presence of thrombin [67]. However, recently, it has been observed that fibrin clot properties determined in patients taking NOACs may provide prognostic information regarding recurrent VTE risk [68,69].

Previously, it has also been shown that metformin added to plasma reduces FXIII-mediated fibrin crosslinking leading to increased plasma clot lysability assessed in vitro [70]. A 7-day aspirin ingestion at a dose of 75 mg/d inhibited FXIII activation due to diminished thrombin generation [71]. Interestingly, aspirin intake was associated with slower FXIII activation in the FXIII 34Leu healthy carriers (both heterozygous and homozygous) compared to non-carriers [71]. Specific inhibitors targeting FXIII, not interfering with thrombin generation, fibrin formation, or with platelet activation are being investigated as a potential anticoagulant therapy [45].

## 5. Conclusions

It is known that FXIII levels and activity influence fibrin clot composition and properties in the acute phase of VTE. A significant decrease in FXIII activity and reduced antigen levels are observed in acute PE and DVT patients, which indicates its consumption in this process. FXIII activity and levels returned to normal values after several weeks since the VTE episode. Although acute PE has been shown to be associated with the prothrombotic fibrin clot phenotype, reduced clot-bound FXIII-A amounts were identified using proteomic analysis in PE patients compared to controls. Interestingly, clot-bound FXIII levels increased after three months of anticoagulant treatment, along with improved fibrin clot properties, but the clot-bound FXIII amounts were still lower than in controls.

Since FXIIIa also crosslinks histones to fibrin, anticoagulant therapy using parenteral heparins can inhibit the histone-fibrin crosslinking and improve fibrinolysis. NOACs and other widely used anticoagulants can also improve fibrin clot properties, increase plasma FXIII activity, and alter clot-bound amounts of FXIII. It has been suggested that FXIIIa inhibitors might be a novel therapeutic option in acute VTE, but clinical studies are needed to confirm their clinical utility. The mechanisms behind FXIII-mediated modulation of fibrin properties in acute VTE are still obscure. Since therapeutic options affecting FXIII levels might be of potential clinical importance in acute VTE patients, the role of FXIII in human thromboembolism deserves further research efforts.
